# Characterization of Food Chain *Clostridioides difficile* Isolates in Terms of Ribotype and Antimicrobial Resistance

**DOI:** 10.3390/microorganisms11051296

**Published:** 2023-05-16

**Authors:** Pilar Marcos, Aoife Doyle, Paul Whyte, Thomas R. Rogers, Máire McElroy, Seamus Fanning, Jesus Frias, Declan Bolton

**Affiliations:** 1 Teagasc Food Research Centre, Ashtown, Dublin 15, D15 KN3K Dublin, Ireland; 2 School of Veterinary Medicine, University College Dublin, Belfield, Dublin 4, D04 V1W8 Dublin, Ireland; 3 Department of Clinical Microbiology, Trinity College Dublin, Central Pathology Laboratory, St James’s Hospital, Dublin 8, D08 RX0X Dublin, Ireland; 4 Central Veterinary Research Laboratory, Department of Agriculture, Food and the Marine, Backweston, Celbridge, W23 X3PH Kildare, Ireland; 5 UCD-Centre for Food Safety, School of Public Health, Physiotherapy and Sports Science, University College Dublin, Belfield, Dublin 4, D04 V1W8 Dublin, Ireland; 6 Environmental Sustainability and Health Institute, Technological University Dublin, Grangegorman, Dublin 7, D07 H6K8 Dublin, Ireland

**Keywords:** *Clostridioides difficile*, ribotype, antibiotic resistance, food, farm, abattoir, retail

## Abstract

The aim of this study was to characterize *C. difficile* isolates from the farm, abattoir, and retail outlets in Ireland in terms of ribotype and antibiotic resistance (vancomycin, erythromycin, metronidazole, moxifloxacin, clindamycin, and rifampicin) using PCR and E-test methods, respectively. The most common ribotype in all stages of the food chain (including retail foods) was 078 and a variant (RT078/4). Less commonly reported (014/0, 002/1, 049, and 205) and novel (RT530, 547, and 683) ribotypes were also detected, but at lower frequencies. Approximately 72% (26/36 tested) of the isolates tested were resistant to at least one antibiotic, with the majority of these (65%; 17/26) displaying a multi-drug (three to five antibiotics) resistant phenotype. It was concluded that ribotype 078, a hypervirulent strain commonly associated with *C. difficile* infection (CDI) in Ireland, was the most frequent ribotype along the food chain, resistance to clinically important antibiotics was common in *C. difficile* food chain isolates, and there was no relationship between ribotype and antibiotic resistance profile.

## 1. Introduction

*Clostridioides difficile* is an anaerobic, Gram-positive, spore-forming bacterium of ubiquitous nature. They have been isolated from the feces of a range of different animals that are both asymptomatic or suffering from severe enteric disease and are the leading cause of antibiotic-associated diarrhea in humans [1]. Symptoms caused by *C. difficile* infection (CDI) vary from mild to serious and fulminant colitis, which may result in mortality [2]. The factors promoting CDI include treatment with antibiotics. Although treatment with nearly all classes of antibiotics has been associated with CDI, third-generation cephalosporins, penicillins, clindamycin, and fluoroquinolones active against the commensal bowel flora are considered to present the highest risk. These anti-bacterial compounds disrupt the gut microbiota, remove competing organisms, and thus facilitate *C. difficile* outgrowth, colonization, and toxin production [3]. 

Ribotyping is a tool used for the definition of lineages and the study of the epidemiology in humans based on the heterogeneity of the ribosomal intergenic spacer region (ISR) [4]. This technique detects the ISR variation of the 11–12 copies within the rRNA operon in the genome. PCR of this region generates fragments of different lengths, which can be identified by capillary gel-based electrophoresis or be compared online against a database of isolates with known ribotypes [5]. 

There are currently over 800 *C. difficile* ribotypes reported worldwide. Only toxin-producing strains cause disease in humans, with several of these having increased virulence (hypervirulence) [6]. Diarrhea is associated with the production of toxin A (TcdA) and toxin B (TcdB), both of which cause intestinal inflammation. Toxigenic strains may also produce *C. difficile* transferase (CDT), a third unrelated binary toxin thought to play a role in epithelial adhesion that is associated with enhanced virulence and higher patient mortality [6,7]. *C. difficile*’s ribotypes can be classified into five main clades (I-V). Among these, Clade II and V are considered the most relevant as they include hypervirulent ribotypes 027 and 078, respectively, and other clinically important RTs, such as RT244 and RT176 [7].

Several different ribotypes have been detected in hospitals and associated with human illness and outbreaks. Ribotypes 014/020 (20.5%), 001 (18.2%), and 078/126 (18.2%), for example, were detected in Spanish hospitals in a study by Alcalá et al. [8]. Ribotypes RT002 (11.6%), RT015 (10.9%), and RT005 (7.2%) were isolated from CDI patients in England [9], and ribotypes 014/020 and 001/072 are considered endemic throughout Europe [10]. In recent years, ribotypes 078 (17%), 020 (9%), and 014 (8%) have been frequently detected in Ireland [11]. 

Although *C. difficile* was historically considered to be a hospital-associated disease, there is a high genetic relatedness between human and animal isolates which often belong to the same genotype [12]. Indeed whole genome sequencing (WGS) analysis has shown that isolates from human, animal, and environmental sources are often indistinguishable [13]. The multi-directional spread of *C. difficile* occurs between these different sources and inevitably results in the contamination of food [14]. 

It is, therefore, to be expected that the same *C. difficile* ribotypes occur in food-producing animals and humans [15,16]. Ribotypes 078 and 126, for example, often isolated from CDI patients, are also common in pigs [12,17], poultry [18], and cattle [19]. Although less information is available for sheep, previously detected ribotypes in humans (RT014, RT010, and RT045) have been isolated from ovine sources [20,21,22]. 

Many of these ribotypes, including 078, 126, 027, and 014/020, have also been isolated from a range of retail foods, including pork, turkey, beef [23,24,25], chicken [18,26], shellfish and bivalve mollusks [27,28,29], and vegetables [30,31,32,33]. 

Antibiotics have played an important role in the emergence of *C. difficile*. Tetracycline usage in animal husbandry, for example, was a key driver in the emergence of hypervirulent RT078, which acquired tetracycline resistance elements from *Streptococcus suis*, *Enterococcus faecalis,* and *Escherichia coli* when under selective pressure [34]. Treatment with antibiotics is also a risk factor for current human infections, which are often associated with the disruption of the gastrointestinal tract microflora [35].

CDI in humans is treated with vancomycin, metronidazole, or a combination of both, which has inevitably increased the prevalence of resistance to these antibiotics in *C. difficile* [36]. Plasmid-mediated resistance to metronidazole was recently identified by Boekhoud et al. [37]. Thus, the current guidelines on the management of *C. difficile* infection in adults in the USA recommend treatment using vancomycin and fidaxomicin instead of metronidazole [38]. Additionally, many *C. difficile* strains are resistant to clindamycin, erythromycin, rifamycins, fluoroquinolones, and penicillins, commonly used to treat other bacterial-associated illnesses in humans [3,39]. The prevalence of *C. difficile* resistance to metronidazole and vancomycin varies in different countries and has been reported to be 3.6–17.9% in the USA and 44.6% in Israel, respectively [40,41]. Considerably lower rates have been described in the UK and Ireland (0.7% metronidazole resistance in the former and 2% vancomycin resistance in Ireland) [42]. Resistance to rifampicin ranges from 0.7% in the UK to 63.6% in Hungary [43,44] and 65.3% in the USA [41,45]. 

The increasing resistance of *C. difficile* to antibiotics has driven the emergence of new ribotypes resulting in altered epidemiology in humans [46]. For example, the rapid emergence of hypervirulent *C. difficile* RT027 during the early 2000s has been attributed to the acquisition of fluoroquinolone resistance, a conjugative transposon in two distinct lineages, and the frequent use of this antibiotic for the treatment of a range of gastrointestinal infections in humans [47]. Despite this link between ribotypes and antibiotic resistance, the majority of studies to date have reported one or the other but not both.

Previous research in our group found *C. difficile* at all stages of the food chain in Ireland and obtained isolates from farms, abattoirs, and retail foods [48]. The objective of the current study was to characterize these isolates in terms of ribotype, with a representative selection being tested for antibiotic resistance.

## 2. Materials and Methods

### 2.1. C. difficile Isolates

The isolates were previously obtained in our laboratory as part of a surveillance study [48]. Isolates tested belonged to bovine farm water (5), bovine farm soil (6), bovine carcass (1), ovine feces (1), ovine carcasses (2), ovine farm soil (4), broiler feces (1), broiler farm water (2), broiler farm soil (11), wild rocket leaves (1), coleslaw (1), and cottage cheese (1).

### 2.2. PCR Ribotyping

The protocol used for PCR ribotyping was that described by ECDC [49], with minor modifications. The intergenic spacer region between the 16S and 23S rRNA genes was initially amplified by PCR using the primers described by Bidet et al. [50]. Briefly, 12.5 µL of HotStarTaq Mastermix (QIAGEN Ltd., Manchester, UK), 0.25 µL of each primer (10 μM), 10 µL RNAse free water (QIAGEN Ltd., Manchester, UK), and 2 µL of the template were added to a MicroAmp^TM^ Optical 96-well reaction plate (Applied Biosystems, Warrington, Cheshire, UK). Sealed PCR plates were then inserted into a Veriti 96-Well Thermal Cycler (Applied Biosystems, Warrington, Cheshire, UK), where the amplification protocol included a denaturation step of 15 min at 95 °C, followed by 30 cycles of 1 min at 94 °C, 1 min at 60 °C and 1 min at 72 °C, and a final extension of 30 min at 72 °C.

After the initial PCR, denaturation of the generated fragments was required before further analysis in an automated sequence and fragment analysis system. For this, 2 µL of the PCR products were added to 9.5 µL of Highly Deionized (Hi-Di) Formamide (Applied Biosystems, Warrington, Cheshire, UK) and 0.5 µL of GeneScan 1200 LIZ Size Standard (Applied Biosystems, Warrington, Cheshire, UK) and denatured for 2 min at 95 °C in a thermal cycler. The plate was cooled down for 10 min in a fridge before the PCR products were analyzed on an ABI 3500 Genetic Analyzer (Applied Biosystems, Warrington, Cheshire, UK) with default settings for POP7 and 50 cm capillary length. The raw data files obtained (*fsa files) from the ABI 3500 Genetic Analyzer were uploaded to the freely available WEBRIBO database (https://webribo.ages.at/; last accessed 1 September 2022) to compare our isolates with profiles stored in the database and confirm the PCR ribotypes [5].

### 2.3. Minimum Inhibitory Concentration (MIC) Testing

The antibiotics metronidazole, vancomycin, erythromycin, clindamycin, moxifloxacin, and rifampicin were selected for MIC testing. Metronidazole is the recommended first-line treatment option for mild-to-moderate CDI, while vancomycin can be used as a substitute or in combination [51]. Rifampicin is used as an adjunctive therapy to CDI [52]; however, its increased use in tuberculosis treatment has been associated with the emergence of rifampicin-resistant *C. difficile* strains [53], linked with recurrent CDI [54]. Moxifloxacin, erythromycin, and clindamycin are not frequently used to treat CDI, but are widely detected in virulent *C. difficile* strains and are important markers for treatment failure and for the spread of the disease in healthcare settings [55,56,57,58].

E-test strips (bioMérieux, Marcy-l’Étoile, France) were used following the protocol described by the manufacturer. In brief, the isolates were recovered from Protect Select Anaerobe Cryobeads (Technical Service Consultants Ltd., Lancashire, UK) and stored at −80 °C by aseptically transferring a single bead to 30 mL of Mueller–Hinton broth (Oxoid, Basingstoke, Hampshire, UK (CM0405)). Isolates were incubated anaerobically in an A35 Anaerobic workstation (Don Whitley, Victoria Works, Yorkshire, UK) at 37 °C for 24 h or until an OD_600nm_= 0.5 (10^8^ CFU mL^−1^) was achieved as measured using a spectrophotometer DeNovix DS-C (DeNovix Inc., Wilmington, NC, USA). A sterile swab (Sparks Lab Supplies, Dublin, Ireland (SW001)) was then used to spread the broth culture on Brucella, vitamin K, and haemin agar (Sigma-Aldrich, Gillingham, UK (B2926-500G)) with 5% defibrinated horse blood (TCS Biosciences Limited, Botolph Claydon, Buckingham, UK (HB034)). Plates were air-dried for 15 min in the anaerobic cabinet before the E-test strips were aseptically placed on top of the agar using sterilized forceps and incubated at 37 °C for 48 h under anaerobic conditions (as described above). The MIC values were then obtained using the scale (µg mL^−1^) provided by the manufacturer. MIC values for each antibiotic were compared to the epidemiological cutoff values (ECOFF) and breakpoints found in the European Committee on Antimicrobial Susceptibility Testing (EUCAST) [59] for *C. difficile* and classified as susceptible or resistant accordingly. 

## 3. Results

The characterization of the isolates in terms of PCR ribotyping and antimicrobial resistance is presented in Table 1. 

In bovines, all of the five isolates from farm water belonged to RT078 (two isolates) or the variant 078/4 (3). One of these was sensitive to all of the antibiotics tested, two were resistant to VAN or MOX, while the remaining isolates displayed multi-resistant phenotypes (MET-VAN-RIF and MET-MOX-VAN-RIF). Ribotypes 078, 078/4, 049, 547, and 683 and an inconclusive result were obtained in bovine farm soil with corresponding antibiotic resistance profiles VAN, ERY-MOX-VAN-RIF, ERY-MET-CLIN-VAN-RIF, MET-VAN-RIF, and sensitive to all and ERY-MET-CLIN-VAN-RIF, respectively. A bovine carcass isolate (RT078/4) was resistant to MET-VAN-RIF. 

The ovine feces isolate was ribotype 078/4 and resistant to MOX. Ovine farm soil isolates belonged to RT078 (three isolates), with one isolate being an inconclusive ribotype and demonstrating three different phenotypical resistance profiles (ERY-CLIN-MOX, MOX, and sensitive to all). Both ovine carcass isolates were RT078/4, and one showed antimicrobial resistance to ERY-MET-VAN. 

In broilers, a fecal isolate (RT078) showed simultaneous resistance against ERY-MET-CLIN-VAN-RIF. In water collected from broiler farms, one of the two isolates was resistant to MOX (RT078/4), while the other was susceptible (inconclusive RT). Ribotypes detected in broiler farm soil (11 isolates) had diverse ribotypes and antibiotic resistance patterns, including RT078 (MET-VAN-RIF and two sensitive), 078/4 (MET-VAN-RIF, MOX-VAN, and MET-MOX, one each), 049 (ERY-MET-CLIN-VAN-RIF), 002/1 (CLIN-MOX-VAN-RIF), 014/0 (ERY-MET-CLIN-VAN-RIF), and 205 and 530, both of which were sensitive to all antibiotics tested.

In retail foods, all strains belonged to RT078, although antibiotic resistance profiles differed. In cottage cheese, susceptibility to all antibiotics tested was reported. In coleslaw and wild rocket leaves, resistance to MET-VAN-RIF and ERY-MET-VAN-RIF was observed, respectively.

Overall, the highest resistance was observed for VAN (52.7%), followed by RIF and MET (41.6% each), MOX (30.5%), ERY (25%), and CLIN (19.4%) and 47% of the isolates were multi-drug resistant (≥three antibiotics). 

## 4. Discussion

### 4.1. C. difficile’s Ribotype Prevalence along the Food Chain

In this study, nine different *C. difficile* ribotypes were detected, which reflects the diversity in sample type and source. RT078 and its variant (RT078/4) were the most common ribotypes. This was not unexpected as several previous studies have reported RT078 to be the predominant ribotype in meat and vegetable food chains [1,18,19,30,60,61,62,63].

The bovine isolates belonged to ribotypes 078, 078/4, 049, 547, and 683. Previous studies also reported ribotype 078 in bovine feces [1,19,60], while other authors have reported ribotype AI-82/1 [64,65], which was not detected in bovine isolates in our study.

The ovine isolates were identified as RT078 and 078/4. To the best of our knowledge, none of these ribotypes have been previously reported in sheep. Other ribotypes found in sheep and not detected in our study include 066 [21], 045 [66], 015, and 097 [67].

Broiler isolates belonged to RT-078, 078/4, 002/1, 014/0, 049, 205, and 530. Of these, ribotypes 002, 049 [68], 014 [64,65,68], and 205 [61,69] had been previously found in broilers. 

The retail isolates included ribotype 078, which has only been previously reported in retail foods [30]. 

Moreover, to the best of our knowledge, this is the first time that ribotypes 530, 547, and 683 have been described in a *C. difficile* study.

### 4.2. Antimicrobial Resistance of the Selected C. difficile Isolates

Our study suggests there is no relationship between a specific ribotype and the antibiotic resistance phenotype. RT078, for example, had several resistance patterns, including susceptibility to all antibiotics (38.5%; 5/13), resistance to VAN (15.4%; 2/13), MET-VAN-RIF (15.4%; 2/13), MOX, ERY-MOX-CLIN, ERY-MET-VAN-RIF, and ERY-MET-CLIN-VAN-RIF (each 7.69%; 1/13). Solomon et al. [70] reported there was no correlation between clinical RT078 isolates and antimicrobial resistance patterns with diverse profiles obtained even in isolates from the same healthcare setting. These findings are consistent with the suggestion by Spigaglia et al. [39] that there are multiple drivers for antibiotic resistance in *C. difficile*, including the acquisition of genetic elements, alterations in target sites, changes in metabolic pathways, etc. Moreover, the same authors reported that antibiotic resistance is maintained in *C. difficile* regardless of the burden imposed on the cell or the absence of a selective pressure. 

The observed resistance to erythromycin (25%) in isolates from bovine (three), ovine (one), and broiler (two) farm soil, ovine and broiler carcasses (one each), and wild rocket leaves (one), has been previously described in isolates from dairy and beef cattle [71,72], sheep [22], and human patients [73,74,75,76]. At the retail level, erythromycin-resistant *C. difficile* has been widely reported in beef, sheep, goat, and poultry meats [69,77], lettuce [78,79], and ready-to-eat foods [80].

Metronidazole is used to treat a range of infections in both food animals and humans, including CDI. A high percentage of isolates were resistant to metronidazole (42%), including broiler feces (one), bovine farm water (two), bovine (three), ovine (one), and broiler (five) farm soil, bovine carcass (one), wild rocket leaves (one), and coleslaw (one). Even though metronidazole resistance was reported to be rare in *C. difficile* [81], metronidazole-resistant *C. difficile* strains have been isolated from cattle [82], chicken, beef, and sheep carcasses [83,84], foods such as meat products [85], and lettuce [79] and humans [39,42,86]. This is often mediated by mutations in *gyrA/B* [81].

One-fifth (20%) of the *C. difficile* strains tested in our study displayed clindamycin resistance. These isolates came from sources such as broiler feces (one), bovines (two), and soil from ovine (one) and broiler (three) farms. *C. difficile*-resistant strains have been previously reported in cattle [87] and poultry [18,65,88], on beef and sheep carcasses [83], in raw beef and cooked pork meats [89,90,91,92], ready-to-eat salads [78,79,80], and human isolates [42,72,74,93]. It has been suggested that clindamycin-resistant *C. difficile* strains possess the *erm(B)* gene, which also confers resistance to erythromycin [57,94]. Our data provide further evidence of these co-resistances in *C. difficile*.

Eleven of the 36 *C. difficile* isolates tested were resistant to moxifloxacin, which included ovine feces (one), bovine (two) and broiler (one) farm water, and bovine (one), ovine (three), and broiler (three) farm soil. Similar results were previously described in *C. difficile* strains from cattle [72,95,96], soil on poultry farms [65], pig carcasses [97], ready-to-eat salads [58,78], and clinical isolates [93,98,99]. Furthermore, reduced susceptibility to moxifloxacin has been linked to increased mortality in human CDI [100].

Approximately half (53%, 19/36) of our isolates displayed vancomycin resistance. These isolates were from broiler feces (one), bovine farm water (three), bovine (five) and broiler (six) farm soil, bovine and ovine carcasses (one each), and wild rocket leaves (one) and coleslaw (one). Other authors have reported vancomycin-resistant *C. difficile* strains in calf feces [87], doner kebab and meatballs [85], and human patients [40,41,42,101]. Vancomycin and metronidazole are commonly used drugs to treat *C. difficile* infection [2], with Adler et al. [40] describing resistance to both antibiotics simultaneously, as observed in this study.

In addition, 15 of the isolates were resistant to rifampicin, which were isolated from broiler feces (one), bovine farm water (two), bovine (four) and broiler (five) farm soil, bovine carcass (one), wild rocket leaves (one), and coleslaw (one). Rifaximin resistance in *C. difficile* isolates is associated with point mutations in the *rpoB* gene which encodes the beta subunit of RNA polymerase [52] and has been previously reported in clinical isolates [52,81,99,100,101,102,103]. Indeed, in recent years there has been increasingly reported rifampicin resistance in *C. difficile*, reflecting the increased use of this antibiotic in medicine [104,105,106].

Multi-drug resistance, defined as resistance to three or more antibiotics simultaneously [107], was detected in 17 out of the 36 isolates tested from a range of stages along the food chain. Among these, the most relevant were two *C. difficile* strains isolated from retail foods resistant to MET-VAN-RIF in coleslaw and ERY-MET-VAN-RIF in wild rocket leaves, similar to that described in lettuce (ERY-MET-VAN) by Han et al. [79]. Multi-drug-resistant bacteria are often present in rinse water, and vegetable products may be cross-contaminated during the washing process [108]. Moreover, in recent years, *C. difficile* strains with multi-drug resistance have been associated with major outbreaks [76,109].

As humans, animals, and the environment are reservoirs for clinically important ribotypes with multi-directional spread, the health of all three is interconnected and reliant on adopting a One Health approach. Thus, a range of different stakeholders must work together to reduce the emergence and spread of antibiotic-resistant *C. difficile* strains. This should include the restricted use of antibiotics in both human and veterinary settings, but more comprehensive control will require the development of vaccines to reduce carriage in both humans and animals [13]. 

## 5. Conclusions

Overall, this study PCR-ribotyped and examined the antibiotic resistance phenotype of 36 food chain isolates. It was concluded that RT078 was the predominant ribotype, although another seven were identified, including three new ribotypes. Resistance to clinically important antibiotics such as erythromycin (25%; 9/36), metronidazole (42%; 15/36), clindamycin (19%; 7/36), moxifloxacin (31%; 11/36), vancomycin (53%; 19/36), and rifampicin (42%; 15/36) was common, with 72% (26/36) of isolates resistant to at least one antibiotic and 47% (17/36) displaying a multi-drug resistant phenotype. These data suggest that virulent ribotypes of *C. difficile* with phenotypic antibiotic resistance are present in the food chain, which could contribute to the spread of the infection to high risk groups in the community. Our data also suggested that there was no association between ribotype and antibiotic resistance profiles.

## Figures and Tables

**Table 1:** PCR ribotyping, antibiotic susceptibility phenotype profiles, and minimum inhibitory concentration (MIC) values of the isolates tested from different stages of the food chain. ERY = Erythromycin (Resistant > 4 µg mL^−1^), MET = Metronidazole (Resistant > 2 µg mL^−1^), CLIN = Clindamycin (Resistant > 16 µg mL^−1^), MOX = Moxifloxacin (Resistant > 4 µg mL^−1^), VAN = Vancomycin (Resistant > 2 µg mL^−1^), and RIF = Rifampicin (Resistant > 0.004 µg mL^−1^) [59]. Resistance to each antibiotic according to the ECOFF values is highlighted in bold.

Isolate Source	RT ^1^	Antimicrobial Susceptibility (S) or Resistance (R) (MIC in µg mL^−1^)					
		ERY	MET	CLIN	MOX	VAN	RIF
Bovine farm water	078	S (1)	S (0.094)	S (4)	S (<0.002)	**R (3)**	S (<0.002)
	078	S (1.5)	S (0.5)	S (<0.016)	S (<0.002)	S (<0.016)	S (<0.002)
	078/4	S (0.75)	S (0.047)	S (<0.016)	**R (>32)**	S (<0.016)	S (<0.002)
	078/4	S (0.75)	**R (>256)**	S (0.032)	S (3)	**R (>256)**	**R (0.5)**
	078/4	S (0.25)	**R (>256)**	S (8)	**R (>32)**	**R (>256)**	**R (0.5)**
Bovine farm soil	078	S (2)	S (0.125)	S (4)	S (<0.002)	**R (>256)**	S (<0.002)
	078/4	**R (8)**	S (0.19)	S (8)	**R (>32)**	**R (>256)**	**R (0.064)**
	049	**R (>256)**	**R (>256)**	**R (>256)**	S (3)	**R (>256)**	**R (1)**
	547	S (0.25)	**R (>256)**	S (0.064)	S (2)	**R (>256)**	**R (1)**
	683	S (0.75)	S (0.125)	S (4)	S (<0.002)	S (2)	S (<0.002)
	IN ^2^	**R (>256)**	**R (>256)**	**R (>256)**	S (1)	**R (>256)**	**R (0.19)**
Bovine carcass	078/4	S (0.5)	**R (>256)**	S (0.047)	S (3)	**R (>256)**	**R (1)**
Ovine feces	078/4	S (<0.016)	S (<0.016)	S (6)	**R (>32)**	S (1.5)	S (<0.002)
Ovine farm soil	078	**R (>256)**	S (0.125)	**R (>256)**	**R (>32)**	S (1)	S (<0.002)
	078	S (<0.016)	S (<0.016)	S (<0.016)	S (<0.002)	S (<0.016)	S (<0.002)
	078	S (1.5)	S (0.047)	S (<0.016)	**R (>32)**	S (<0.016)	S (<0.002)
	IN^2^	S (<0.016)	S (<0.016)	S (8)	**R (>32)**	S (0.75)	S (<0.002)
Ovine carcasses	078/4	**R (24)**	**R (>256)**	S (6)	S (0.064)	**R (3)**	S (0.003)
	078/4	S (1.5)	S (0.047)	S (2)	S (0.5)	S (1.5)	S (<0.002)
Broiler feces	078	**R (>256)**	**R (>256)**	**R (>256)**	S (3)	**R (>256)**	**R (0.19)**
Broiler farm water	078/4	S (<0.016)	S (<0.016)	S (<0.016)	**R (>32)**	S (<0.016)	S (<0.002)
	IN ^2^	S (<0.016)	S (<0.016)	S (<0.016)	S (<0.002)	S (<0.016)	S (<0.002)
Broiler farm soil	078	S (<0.016)	S (<0.016)	S (<0.016)	S (0.75)	S (<0.016)	S (<0.002)
	078	S (0.38)	**R (>256)**	S (0.064)	S (2)	**R (>256)**	**R (0.5)**
	078	S (<0.016)	S (<0.016)	S (<0.016)	S (<0.002)	S (<0.016)	S (<0.002)
	078/4	S (1.5)	**R (>256)**	S (<0.016)	S (3)	**R (>256)**	**R (1)**
	078/4	S (1)	S (0.047)	S (8)	**R (>32)**	**R (3)**	S (<0.002)
	078/4	S (1)	**R (>256)**	S (6)	**R (>32)**	S (<0.016)	S (<0.002)
	049	**R (>256)**	**R (>256)**	**R (>256)**	S (1.5)	**R (>256)**	**R (2)**
	002/1	S (0.25)	S (<0.016)	**R (>256)**	**R (>32)**	**R (>256)**	**R (0.75)**
	014/0	**R (>256)**	**R (>256)**	**R (>256)**	S (1.5)	**R (>256)**	**R (0.75)**
	205	S (0.25)	S (<0.016)	S (6)	S (<0.002)	S (<0.016)	S (<0.002)
	530	S (<0.016)	S (<0.016)	S (<0.016)	S (0.75)	S (<0.016)	S (<0.002)
Coleslaw	078	S (0.5)	**R (>256)**	S (0.5)	S (2)	**R (>256)**	**R (1)**
Cottage cheese	078	S (0.19)	S (<0.016)	S (<0.016)	S (0.5)	S (<0.016)	S (<0.002)
Wild rocket leaves	078	**R (>256)**	**R (>256)**	S (0.25)	S (1.5)	**R (>256)**	**R (0.047)**
% Resistance		25 (9/36)	41.6(15/36)	19.4 (7/36)	30.5 (11/36)	52.7 (19/36)	41.6 (15/36)

^1^ RT = Ribotype; ^2^ IN = Inconclusive result.

## Data Availability

Data available upon request.
